# Sirt1/Nrf2 pathway is involved in oocyte aging by regulating Cyclin B1

**DOI:** 10.18632/aging.101609

**Published:** 2018-10-27

**Authors:** Rujun Ma, Wei Liang, Qin Sun, Xuhua Qiu, Ying Lin, Xie Ge, Kadiliya Jueraitetibaike, Min Xie, Ji Zhou, Xuan Huang, Qiang Wang, Li Chen, Bing Yao

**Affiliations:** 1 Center of Reproductive Medicine, Jinling Hospital, Clinical School of Medical College, Nanjing University, Jiangsu, People's Republic of China; 2 Traditional Chinese Medicine Department, Nanjing No.454 Hospital, Jiangsu, People's Republic of China; 3 State Key Laboratory of Reproductive Medicine, Nanjing Medical University, Jiangsu, People's Republic of China; * Equal contribution

**Keywords:** Nrf2, Sirt1, oocyte meiosis, oocyte aging, spindle organization

## Abstract

Nuclear factor erythroid 2-related factor 2 (Nrf2) is capable of inducing a variety of biological effects, and the regulation of the Nrf2 signaling pathway is closely related to longevity. To find out whether the nuclear factor erythroid 2-related factor 2 (Nrf2) is involved in oocyte aging or not which may cause reduced female fertility, a series of biological methods was applied, including oocyte collection and culture, micro injection, RNA interference, western blotting, immunofluorescence and confocal microscopy, and quantitative real-time PCR.Our data demonstrated that Nrf2 depletion disrupted oocyte maturation and spindle/chromosome organization by suppressing Cyclin B1 expression. Sirtuin 1 (Sirt1) depletion reduced Nrf2 expression, which indicated the existence of the Sirt1-Nrf2-Cyclin B1 signaling pathway in mouse oocytes. Additionally, immunoblotting results reflected a lower Nrf2 protein level in oocytes from aged mice, and maternal age-associated meiotic defects can be ameliorated through overexpression of Nrf2, which supported the hypothesis that decreased Nrf2 is an important factor contributing toward oocyte age-dependent deﬁcits. Furthermore, we show that the expression of Nrf2 is related to female age in ovarian granular cells, suggesting that the decreased expression of Nrf2 may be related to the decline in the reproductive capacity of older women.

## Introduction

Oocyte quality is a critical factor of female fertility, which can be affected by age. Advanced reproductive biotechnologies depend on a sufficient source of oocytes. In mammals, oocytes are initiated during fetal development and arrested at the germinal vesicle (GV) stage. Fully grown oocytes resume meiosis after stimulation by luteinizing hormone at puberty to reach the second meiotic division, and then arrest at metaphase of meiosis II (MII) until fertilization [1,2]. The process from GV to MII includes a complex sequence of nuclear and cytoplasmic events that prepare the oocyte for fertilization and initiation of embryo development, including accurate control of spindle assembly and chromosome organization [3]. The incidence of aneuploidy increases with age [4]. Although the molecular biology of oocyte meiosis has been proposed to contribute toward age-associated deficits in oocyte meiosis, the mechanisms that modulate the meiotic apparatus remain to be discovered. Sirtuins have been widely reported to be involved in multiple biological processes. Lines of studies have shown that Sirtuin1 (Sirt1) is involved in transcriptional regulation, chromatin modiﬁcation, energy metabolism and aging [5-7]. Increased Sirt1 activity could counteract age-related systems impairment [8]. Moreover, Sirt1 signaling protects mouse oocytes against oxidative stress during aging [9]. It has also been reported that Sirt1 is associated with the activation of nuclear factor-E2 related factor 2 (Nrf2) [10]. As an important transcription factor, Nrf2 has been recognized as a crucial transcription factor that mediates protection against oxidants and enhances cell survival in many tissues [11]. To date, Nrf2 has been linked to the regulation of mitotic progression, especially timely M phase entry [12], and Nrf2 deficiency has been reported to cause a delay in maternal hepatocyte proliferation, concomitant with dysregulation of the activation of Cyclin D1, E1 and A2 [13].

Based on the aforementioned information, we hypothesized that Nrf2, regulated by Sirt1, plays an important role in oocyte aging. . . By investigating the role of Sirt1 and Nrf2 in mouse oocyte we discovered the manipulation of Sirt1 on Nrf2 and the involvement of Nrf2 in the regulation of spindle/chromosome organization and cell division during oocyte aging, and report our ﬁndings here.

## RESULTS

### Reduced Nrf2 expression is detected in aged mouse oocytes

**Figure 1:**
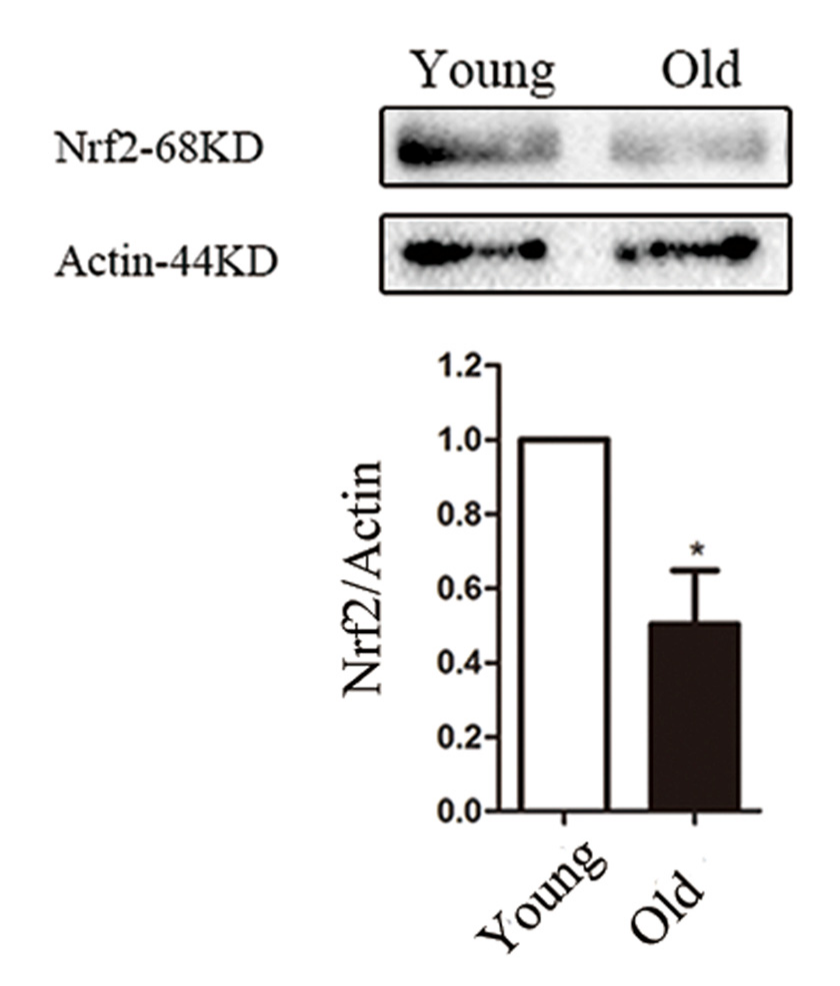
**Nrf2 decline in old mouse oocytes.** Western blot analysis revealed a reduced Nrf2 expression in mouse oocytes from aged females compared with those from young controls. Actin served as a loading control throughout. Band intensity was calculated using ImageJ software, the ratio of Nrf2/Actin expression was normalized and values are indicated. Data are expressed as the mean ± SD, *P<0.05 vs. control.

Transcription factor Nrf2 is a key regulator of the antioxidant defense system, aging-associated diseases and inflammation [14,15]. Therefore, we checked whether Nrf2 expression in oocytes was accordingly changed in response to maternal age. The Nrf2 protein levels in young oocytes (isolated from 6-8 week mice) and old oocytes (isolated from 8-10 month mice) were compared, and a decrease in the Nrf2 level was detected in the old oocytes (P < 0.05; Fig. 1), suggesting that such a decrease may contribute toward the occurrence of observed meiotic defects in old oocytes.

### Cellular distribution of Nrf2 during oocyte meiosis

**Figure 2:**
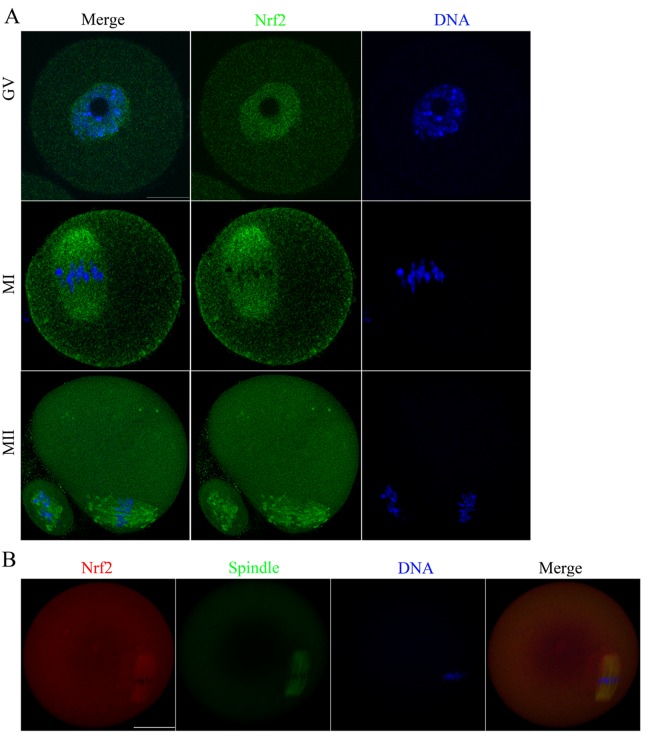
**Cellular distribution of Nrf2 during oocyte meiosis.** (**A**) Immunofluorescent staining was employed to show the subcellular localization of Nrf2. Green, Nrf2; Blue, chromatin. A total of 30 oocytes were examined for each group. Scale bar, 20 μm. (**B**) Immunoﬂuorescent staining for co-localization of α-tubulin with Nrf2 in mouse oocytes. This shows the co-localization of Nrf2 with spindles in mouse oocyte. Red, Nrf2; green, tubulin; blue, chromatin. Scale bar, 20 μm.

To explore the potential involvement of Nrf2 in oocyte maturation, we ﬁrst examined Nrf2 distribution at different developmental stages (Fig. 2A). Immunostaining clearly showed that Nrf2 was expressed in mouse oocyte. The fluorescence signals reside in the entire immature oocytes, and appear to be accumulated in the germinal vesicles. When the oocytes enter metaphase, Nrf2 localized around the spindle region in the course of spindle formation. During MII, Nrf2 continued to associate with the spindle region. Using a double staining method, we conﬁrmed the co-localization of Nrf2 and α-tubulin (Fig. 2B). Such a dynamic distribution pattern suggested that Nrf2 may have a function in the formation or stability of meiotic spindle, or in the regulation of meiotic progression.

### Nrf2 depletion adversely affects maturational progression in mouse oocytes.

**Figure 3:**
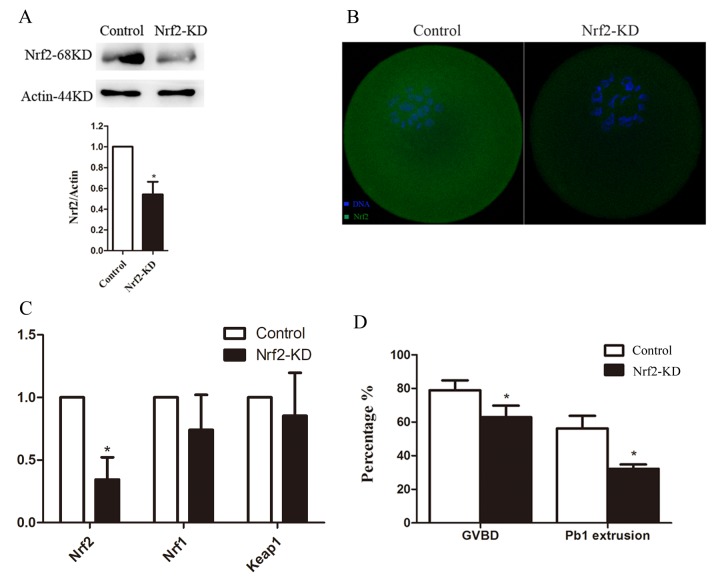
**Effects of Nrf2 knockdown on oocyte maturation**. Fully-grown oocytes injected with Nrf2-siRNA were arrested at the GV stage with milrinone for 20 hours, and were then cultured in milrinone-free medium to evaluate the maturational progression. Negative control siRNA was injected as a control. (**A**) Western blot analysis showing the reduced expression of Nrf2 after siRNA injection. (**B**) Immunostaining showing the loss of Nrf2 in oocytes with siRNA injection. Scale bar, 20 μm. (**C**) The relative mRNA level of Nrf2, Nrf1 and Keep1 were determined by RT-qPCR in control- and Nrf2-siRNA-injected oocytes. mRNA levels in control oocytes were set to 1. (**D**) Quantitative analysis of the GVBD rate and Pb1 extrusion rate in control and Nrf2-siRNA oocytes. Data are expressed as the mean ± SD, *P<0.05 vs. control.

To investigate the function of Nrf2 in oocyte meiosis, we injected Nrf2 siRNA into fully grown oocytes. Then the oocytes were arrested at the GV stage by milrinone treatment for 20 hours to promote mRNA degradation. This led to a signiﬁcant reduction in the expression of both Nrf2 mRNA (Fig. 3C) and protein (Fig. 3A and B) levels (P < 0.05), while other gene products were not directly aﬀected. We then analyzed how Nrf2-knockdown (Nrf2-KD) aﬀects oocyte maturation (Fig. 3C).

After culturing for 3 hours, the germinal vesicle breakdown (GVBD) rate decreased in the Nrf2-knockdown group compared with that in the control group (P < 0.05; Fig. 3D). Moreover, the ratio of first polar body (Pb1) extrusion was significantly reduced (P < 0.05) in Nrf2-KD oocytes after 14-hour in vitro maturation (Fig. 3D). GVBD means oocytes entry M phase, and the extrusion of Pb1 marks the oocyte maturation. These results, similar as our previous study [16], indicate that Nrf2 participates proper meiotic divisions.

### Nrf2 is essential for spindle organization and chromosome alignment in oocytes

**Figure 4:**
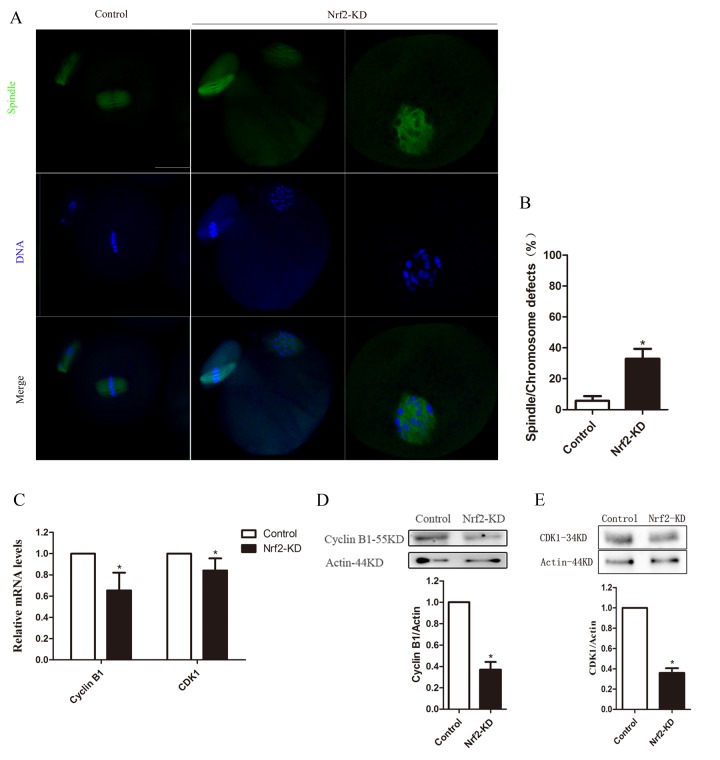
**Effects of Nrf2 knockdown on spindle structure, chromosome alignment, Cyclin B1 and CDK1 levels of mouse oocytes.** (**A**) Control and Nrf2-KD oocytes were collected and stained for visualizing spindle (green) and chromosomes (blue). Scale bar, 20 μm. (**B**) Quantiﬁcation of control and Nrf2-KD oocytes with spindle defects or chromosome misalignment. (**C**) The relative mRNA level of Cyclin B1 and CDK1 were determined by RT-qPCR in control and Nrf2-siRNA injected oocytes. mRNA levels in control oocytes were set to 1. (**D**) Western blot analysis showing the reduced expression of Cyclin B1 after Nrf2 siRNA injection. (**E**) Decreased CDK1 protein levels in Nrf2-KD oocytes. Data are expressed as the mean ± SD, ^*^P<0.05 vs. control.

The above data suggested a possibility that Nrf2 depletion may affect meiotic apparatus in oocytes. To investigate the regulatory mechanism of Nrf2 during meiosis, Nrf2-KD and control oocytes were immunolabeled with anti-tubulin antibody to visualize the spindle and were co-stained with Hoechst 33342 to bind chromosomes. As shown in Fig 4A, most control oocytes at the metaphase stage showed a typical barrel-shaped spindle and well-organized chromosomes at the equator plate. By contrast, oocytes depleted of Nrf2 displayed a high percentage of spindle/chromosome defects, including loose spindles and chromosomal alignment, compared with controls (P < 0.05). These findings imply that the correct assembly of meiotic apparatus in mouse oocytes depends on Nrf2 expression.

### Nrf2 depletion interferes with Cyclin B1/CDK1 expression in mouse oocytes

The effects of Nrf2-KD on meiotic progression prompted us to consider the possible target in mouse oocytes. Therefore, we further analyzed the expression of Cyclin B1 and Cyclin-dependent kinase 1 (CDK1) by qPCR and western blot analysis. The qPCR results shown in Fig. 4C indicated declined Cyclin B1 and CDK1 mRNA levels in Nrf2-KD oocytes (P < 0.05). While there are no significant difference in the expression of Wee1, CDC25A and MYT1, which have been reported can regulate the expression of cyclinB1/CDK1 [17-19], between control and Nrf2-KD oocytes (Fig. S3B). Furthermore, immunoblot analysis showed that the expression of Cyclin B1 and CDK1 proteins in the Nrf2-KD group was significantly reduced (P < 0.05) compared with those in the control group (Fig. 4D-E). The relative protein intensity analysis also confirmed this (loading control: β-actin). Taken together, these results demonstrated that Nrf2 promotes spindle formation and meiotic division in oocytes through regulating Cyclin B1/CDK1.

### Sirt1 depletion alters Nrf2 expression in oocytes

**Figure 5:**
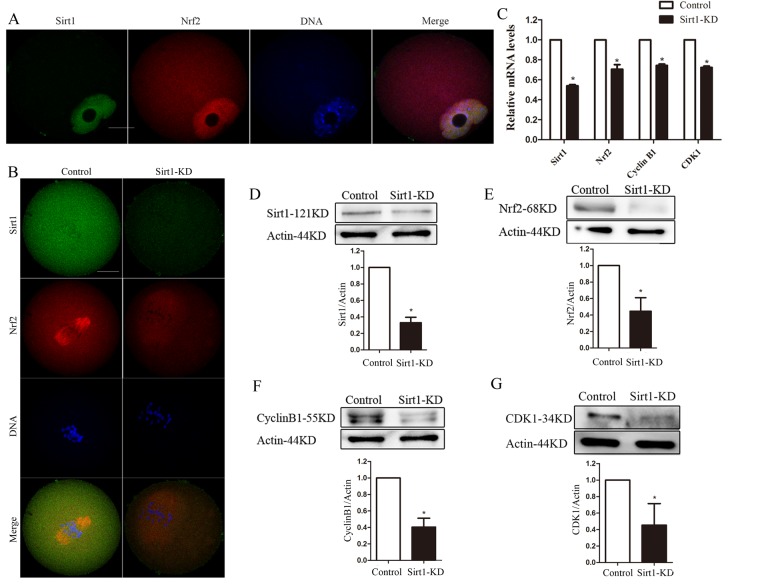
**Sirt1 depletion reduces Nrf2 expression in oocytes.** (**A**) Double staining of GV oocytes with Nrf2 antibody (red) and Sirt1 antibody (green), and counterstaining of chromosome with Hoechst 33342 (blue), conﬁrming the Sirt1 co-localization with Nrf2. (**B**) Control and Sirt1-KD oocytes were double stained with anti-Sirt1 antibody (green) and Nrf2 antibody (red), and counterstained for chromosomes (blue). (**C**) The relative mRNA levels of Sirt1, Nrf2, Cyclin B1 and CDK1 were determined by RT-qPCR in control- and Sirt1-siRNA-injected oocytes. mRNA levels in control oocytes were set to 1. (**D**) Reduced Sirt1 levels after Sirt1-siRNA injection were conﬁrmed by western blot analysis. Actin served as a loading control. Band intensity was calculated using ImageJ. Bars represent the mean ± SD. ^*^P<0.05 vs. control. (**E**-**G**) Western blot analysis showed the reduced Nrf2, Cyclin B1 and CDK1 protein levels in oocytes following Sirt1 knockdown, with actin as a loading control. Bars represent the mean ± SD. ^*^P<0.05 vs. controls.

To identify a signaling pathway that might account for the maintenance of spindle assembly and meiotic division by Nrf2 in oocytes, we assessed the effects of Sirt1 depletion on Nrf2 levels. For this purpose, we ﬁrst examined the localization/levels of endogenous Sirt1 by immunoﬂuorescence. Double staining was also performed in GV oocytes and it was conﬁrmed that Sirt1 co-localized with Nrf2 (Fig. 5A).

**Figure 6:**
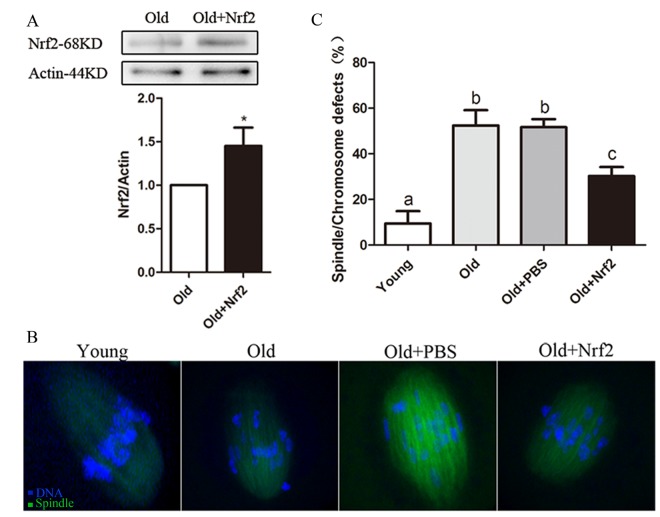
**Maternal age-associated oocyte spindle and chromosome abnormalities suppressed by Nrf2 overexpression.** (**A**) PBS (control group) or Nrf2 plasmid (overexpression group) was microinjected into old GV oocytes, which were arrested for 20 h with milrinone to allow synthesis of new Nrf2 protein. Results indicated that Nrf2 protein was efﬁciently overexpressed. (**B**) Representative examples of meiotic spindle and chromosomes at MII stage in young oocytes, old oocytes and old oocytes injected with PBS or Nrf2 plasmid. Arrowheads indicate misaligned chromosomes. (**C**) Incidence of spindle/chromosome defects in indicated oocytes. Data are expressed as the mean ± SD percentage from 3 independent experiments in which 100 oocytes were analyzed, *P<0.05 vs. control.

Then we injected Sirt1 siRNA into fully grown oocytes, which were arrested at the GV stage by milrinone treatment for 20 hours to promote mRNA degradation. As shown in Fig. 6C, the abundance of Sirt1 mRNA was specifically (p<0.05) decreased. A significant reduction of Sirt1 protein level in oocytes was confirmed by immunofluorescence and western blot analysis (p<0.05; Fig. 5B, D).

Notably, following Sirt1-knockdown, Nrf2 was signiﬁcantly decreased, based on immunostaining and western blotting (Fig. 5B and D). These data suggested that the expression of Nrf2 during oocyte meiosis is regulated by Sirt1. Due to the limited number of oocytes, we examined the relationship between sirt1 and Nrf2 in ovarian tissues as an alternative. CO-IP was performed to confirm the interaction relationship between Sirt1 and Nrf2 in mouse ovaries (Fig. S1). Additionally, compared with controls, immunoblot and quantitative analysis showed that Sirt1-knockdown in oocytes had marked effects on Cyclin B1 and CDK1 expression (P < 0.05; Fig. 5C, E and F). This suggested that Sirt1 may act as the upstream molecule for Nrf2-Cyclin B1/CDK1 in mouse oocytes.

### Nrf2 overexpression ameliorates maternal age-associated oocyte meiotic defects

It has been well documented that female fertility decreases with advanced maternal age due to chromosomal and spindle abnormality in oocytes. We performed overexpression experiments to test whether enhancing Nrf2 expression in old oocytes could rescue their meiotic phenotypes. Nrf2 CRISPR Activation Plasmid was injected into old GV oocytes, which were arrested for 20 h with milrinone to allow synthesis of new Nrf2 protein. Immunoblotting with an anti-Nrf2 antibody conﬁrmed that Nrf2 protein was efficiently overexpressed (P < 0.05; Fig. 6A). Confocal analysis revealed that only 9.4±5.4% of ovulated MII oocytes obtained from young mice exhibited abnormal spindle formation and chromosome alignment; however, 52.3±6.8% of MII oocytes retrieved from aged mice showed spindle defects or misaligned chromosomes. Notably, these abnormalities were only detected in 30.2±4.0% of old oocytes with Nrf2 overexpression, which is signiﬁcantly decreased as compared with old oocytes injected with PBS (51.6±3.5%, P < 0.05; Fig. 6B and C). Taken together, these results suggested that Nrf2 overexpression reduces the occurrence of maternal age-associated meiotic defects.

### Nrf2 is related to age in female granulosa cells

**Figure 7:**
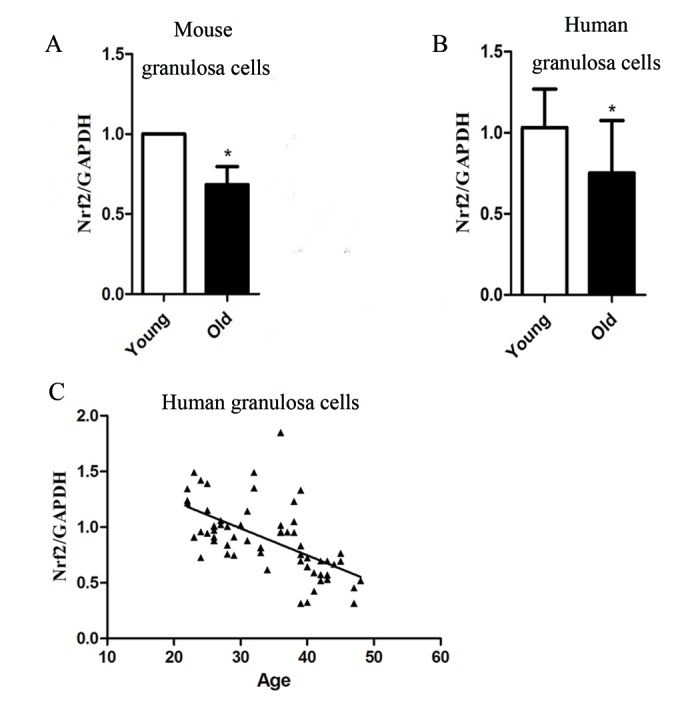
**The correlation between Nrf2 and age in granular cells.** (**A**) The relative mRNA levels of Nrf2 were determined by RT-qPCR in control and old granular cells. mRNA levels in control granular cells were set to 1. Data are expressed as the mean ± SD. (**B**) The relative mRNA level of Nrf2 was determined by RT-qPCR in young (22-35 years) and old (36-49 years) ovarian granular cells. Data are expressed as the mean ± SD, *P<0.05 vs. young group. (**C**) Analysis of correlation between Nrf2 expression and age in human ovarian granular cells. R=-0.5972.

To investigate the consistency of expression trends of Nrf2 in oocytes and granulosa cells, we collected granulosa cells from old and young mice. The result showed the Nrf2 level in old mouse granular cells decreased (Fig. 7A).

Then, we measured the mRNA level of Nrf2 in ovarian granular cells (from donors aged 22-49 years) by quantitative analysis. A signiﬁcant decrease in Nrf2 with age was detected, as observed in mouse granular cells (Fig. 7B). The results showed that the expression of Nrf2 was correlated with age in females (r=-0.5972, P < 0.05), and Nrf2 mRNA levels in the young group were significantly higher than those in the old group (P < 0.05; Fig. 7C). The above results suggested that the decreased expression of Nrf2 may be related to the decline of reproductive capacity of older women.

## DISCUSSION

The studies presented here were designed to investigate the functions of Nrf2 during oocyte meiosis. We found spindle defects and chromosome misalignment in mouse oocytes with Nrf2-knockdown. Moreover, we discovered that Nrf2 controls Cyclin B1/CDK1 levels in oocytes, which are critical for proper cell division maintaining. In addition, our results suggested that the Sirt1-Nrf2-Cyclin B1/CDK1 signaling pathway was important for oocyte meiosis. Finally, we have provided evidence that Nrf2 is a key factor impacting oocyte aging.

It has been found that Nrf2 may be a long-lived gene, and that the regulation of Nrf2 signaling pathway is closely related to longevity [20]. Our data showed that the Nrf2 protein levels declined in aged oocytes, which is in accordance with results in mitotic cells [21]. As reported by others, decline in transcriptional activity of Nrf2 causes age-related loss of glutathione synthesis [22], and age-dependent Nrf2 dysfunction causes decreasing somatic proteasome expression during aging [23]. Crosstalk between Nrf2 and the proteasome suggest a therapeutic potential of Nrf2 inducers in vascular disease and aging [24]. Additionally, Hu et al. [25] found that Nrf2-KO mice had decreased fertility and premature ovarian failure. The deficiency of the transcription factors Nrf2 and Nrf1 could lead to early embryonic lethality [26]. Taken together, these results indicated that Nrf2 may be involved in the aging process and may have certain biological functions.

In the present study, we observed an enrichment of Nrf2 protein on the meiotic apparatus, presumably due to its function in meiotic divisions. Furthermore, as revealed by our knockdown and overexpression experiments, we showed that Nrf2 ablation led to high frequency of chromosome misalignment in these oocytes, which is likely mediated through the Cyclin B1/CDK1 pathway. As previously reported, overexpression of Nrf2 inhibited the PBK/TOPK KD-induced decrease in Cdc2 and Cyclin B expression and cell cycle arrest, and blocked ROS production and apoptosis [27]. Moreover, Cyclin B1 synthesis and degradation regulated maturation-promoting factor (MPF; cyclin-dependent kinase 1/cyclin B1) activity, which oscillates with oocytes entry and exit from meiosis I and meiosis II in mammalian oocytes [28,29]. Our previous study found that Nrf2 overexpression increased the expression of Cyclin B1 in mouse oocytes, which further demonstrated that Nrf2 regulates Cyclin B1 [16]. Here, we revealed that Nrf2 modulates the Cyclin B1 and CDK1 levels in mouse oocytes and, correspondingly, spindle disorganization was detected in Nrf2-KD oocytes (Fig. 4). We found that Nrf2 knockdown change the expression of HO-1, NQO-1 and GCLM, not Wee1, CDC25A and MYT1, suggesting the decline of antioxidant activity may be related to the decrease of Cyclin B/CDK1 which needs to be studied in the further.

Sirtuins are nicotinamide adenine dinucleotide (NAD)-dependent deacetylases that have been reported to be able to relieve from age-inducing stress [30]. Activated Sirt1 has been reported to ameliorate age-related changes, such as menopausal syndrome [31]. Several studies have reported that Sirt1 might be involved in oocyte aging by regulating the redox state and ensuring normal spindle assembly [9,32,33]. In addition, the long-lived gene Sirt1 can regulate the expression and activity of Nrf2 by down-regulating βTrCP [34], and play an important physiological role by activating Nrf2 [35,36]. In the present study, abolishing endogenous Sirt1 resulted in signiﬁcantly reduced Nrf2 expression. Thus, Nrf2 might be a downstream effector of Sirt1 for regulating Cyclin B1 and spindle assembly during meiosis.

The expression of Nrf2 detected in cumulus cells might be related to oocyte quality [37]., while upregulation of Nrf2 in oocytes and cumulus cells might affect the GSH level in matured COCs [38]. Notably, we found Nrf2 decline with age in both mouse oocytes and granulosa cells. Therefore, we hypothesized that Nrf2 decline in the granulosa cells may prompt Nrf2 levels in oocytes to also decrease, resulting in a decline in oocyte quality. Here, our data showed that Nrf2 expression in granulosa cells decreased with age, suggesting that Nrf2 may be related to the decline in oocyte quality in older women, and providing a possible clinical diagnostic indicator. The effect of changed Nrf2 expression in granulosa cells on oocyte quality remains to be further studied.

In conclusion, our data indicated a role for Nrf2 during oocyte maturation and uncovered the striking beneﬁcial effects of Nrf2 overexpression on aged oocytes, which opens a new area for understanding mechanisms as well as assessing oocyte quality. Furthermore, we provided novel evidence showing that Nrf2 could catalyze spindle assembly in oocytes via the Sirt1-Nrf2-Cyclin B1 signaling pathway (Fig. S2).

## MATERIALS AND METHODS

All chemicals and culture media were purchased from Sigma-Aldrich; Merck KGaA (Darmstadt, Germany) unless stated otherwise. ICR mice were used in this study. All animal experiments were approved by the Animal Care and Use Committee of Nanjing Jinling Hospital and were performed in accordance with institutional guidelines.

### Antibodies

Rabbit polyclonal anti-Nrf2 (cat. no., ab137550), mouse monoclonal anti-Sirt1 (cat. no., ab110304), rabbit monoclonal anti-Cyclin B1 (cat. no., ab181593) and rabbit monoclonal anti-CDK1 (cat. no., ab32384) were purchased from Abcam (Cambridge, MA, USA); mouse monoclonal anti-α-tubulin-FITC antibody (cat. no., 76074) was purchased from Sigma-Aldrich; Merck KGaA; FITC-conjugated goat anti-rabbit IgG was purchased from Thermo Fisher Scientific, Inc. (Waltham, MA, USA).

### Granular cells collection

This human study was approved by the Ethics Committee of Nanjing Jinling Hospital, and was performed in accordance with National and International guidelines. A total of 62 Chinese females were recruited (aged 22-49 years). Females with known karyotype abnormalities, previous chemotherapy or radiotherapy, ovarian surgery or autoimmune diseases were excluded. Granulosa cells were derived from the part of the ovaries that had been discarded after routine ovulation. Before initiating the study, written informed consent was obtained from the all participants.

6-8-week-old female ICR mice were injected with human chorionic gonadotrophin (hCG), approximately 46-48 h after injection of 5 IU Pregnant Mares Serum Gonadotropin (PMSG). After 12 h, cumulus-oocyte complex (COC) were harvested from oviduct ampullar, oocytes were removed by hyaluronidase. Granulosa cells were collected for the following assays.

### Oocyte collection and culture

Approximately 46-48 h after injection of 5 IU PMSG, fully-grown immature oocytes were harvested from the ovaries of 6-8-week-old female ICR mice. Enclosed cumulus cells were removed by repeatedly pipetting, and then oocytes were cultured in M2 medium under mineral oil at 37˚C in a 5% CO_2_ incubator. At appropriate time points, oocytes were selected for the following assays.

### siRNA-knockdown and overexpression

Microinjection of siRNA or plasmid was used to knock down Nrf2/Sirt1 or to overexpress Nrf2 with a Narishige microinjector. The Nrf2-siRNA and Sirt1-siRNA sequences are shown in Table S1.

For knockdown experiments, siRNA was diluted with water to give a stock concentration of 30 mM, and then 2.5 picoliter solution was injected into the cytoplasm of fully-grown immature oocytes. A siRNA negative control was injected as a control. For overexpression experiments, Nrf2 CRISPR Activation Plasmid was diluted with water to give a stock concentration of 30 ng/μL, and then 2.5 picoliter solution was injected into oocytes. PBS was injected as a control.

After injections, oocytes were arrested at the GV stage with 2.5 µM milrinone for 20 hours, and were then cultured in milrinone-free M2 medium for maturation.

### Quantitative real-time PCR

Total RNA was isolated from granulosa cells or oocytes using an RNA Isolation Kit (cat. no., KIT0204; Invitrogen; Thermo Fisher Scientific, Inc.), and cDNA was quantified by RT-qPCR using a Roche Light Cycler 96 Real-time PCR system (Roche Diagnostics, Basel, Switzerland). The fold-change in gene expression was calculated using the 2^-∆∆Ct^ method with the house keeping gene, GAPDH, as the internal control. Primer sequences are listed in Table S2.

### Western blotting

Approximately 100 oocytes were lysed in Laemmli sample buffer containing protease inhibitor, and boiled for 5 min before being subjected to 10% SDS-PAGE. A PVDF membrane was used to transfer the separated proteins, and was then blocked in TBST (TBS containing 0.1% Tween 20) and 5% skimmed milk for 1 hour. Then the PVDF membrane was separated and incubated overnight at 4˚C with the following primary antibodies: Rabbit anti-Nrf2 antibody (1:1000), anti-Sirt1 antibody (1:750), anti-Cyclin B1 antibody (1:1000), anti-CDK1 antibody (1:1000) and anti-actin antibody (1:2000). After being washed 3 times in TBST, the membranes were incubated with HRP-conjugated secondary antibodies for 1 hour at room temperature, prior to being processed using an ECL Plus Western Blotting Detection System. ImageJ software (National Institutes of Health, Bethesda, MD, USA) was used to quantify fluorescence intensity as previously described [39,40].

### Immunofluorescence and confocal microscopy

For staining of Nrf2 and Sirt1, oocytes were fixed with 4% paraformaldehyde for 30 minutes and then permeabilized with 0.5% Triton X-100 for 20 minutes. After 1 hour of blocking in 1% BSA-supplemented PBS, samples were incubated overnight at 4˚C with the following primary antibodies: Anti-Nrf2 antibody, anti-Sirt1 antibody and FITC-conjugated anti-tubulin antibody. For tubulin immunofluorescence staining, GV oocytes were extracted for 10 min with 0.1% Triton X-100 before ﬁxation, and then stained with the anti-tubulin antibody. After three washes in PBS, oocytes were labeled with Alexa Fluor 488 goat-anti mouse IgG at room temperature for 1 hour. Hoechst 33342 (blue) was used for chromosome staining. Oocyte samples were mounted on anti-fade medium (Vectashield, Burlingame, CA, USA), and were then examined under a Laser Scanning Confocal Microscope (LSM 710, Zeiss, Germany) equipped with the x40 objectives.

### Statistical analysis

Statistical analysis was performed using SPSS 19.0 software (IBM Corp., Armonk, NY, USA). Correlations were assessed using Pearson’s method. Data are presented as the mean ± standard deviations (SD), unless otherwise indicated. The mean and SD of the data were calculated and statistically analyzed by Student’s t-test and analysis of variance (ANOVA) when appropriate (P<0.05).

## Supplementary Material

Supplementary Figures

Supplementary Tables

## References

[1] Wang Q, Sun QY. Evaluation of oocyte quality: morphological, cellular and molecular predictors. Reprod Fertil Dev. 2007; 19:1–12. 10.1071/RD06103 17389130

[2] Eppig JJ, O’Brien M, Wigglesworth K. Mammalian oocyte growth and development in vitro. Mol Reprod Dev. 1996; 44:260–73. 10.1002/(SICI)1098-2795(199606)44:2<260::AID-MRD17>3.0.CO;2-6 9115726

[3] Schuh M, Ellenberg J. Self-organization of MTOCs replaces centrosome function during acentrosomal spindle assembly in live mouse oocytes. Cell. 2007; 130:484–98. 10.1016/j.cell.2007.06.025 17693257

[4] Jones KT. Meiosis in oocytes: predisposition to aneuploidy and its increased incidence with age. Hum Reprod Update. 2008; 14:143–58. 10.1093/humupd/dmm043 18084010

[5] Rodgers JT, Lerin C, Haas W, Gygi SP, Spiegelman BM, Puigserver P. Nutrient control of glucose homeostasis through a complex of PGC-1alpha and SIRT1. Nature. 2005; 434:113–18. 10.1038/nature03354 15744310

[6] Davoren JB, Kasson BG, Li CH, Hsueh AJ. Specific insulin-like growth factor (IGF) I- and II-binding sites on rat granulosa cells: relation to IGF action. Endocrinology. 1986; 119:2155–62. 10.1210/endo-119-5-2155 2945713

[7] Vaquero A, Scher M, Erdjument-Bromage H, Tempst P, Serrano L, Reinberg D. SIRT1 regulates the histone methyl-transferase SUV39H1 during heterochromatin formation. Nature. 2007; 450:440–44. 10.1038/nature06268 18004385

[8] Ferrara N, Rinaldi B, Corbi G, Conti V, Stiuso P, Boccuti S, Rengo G, Rossi F, Filippelli A. Exercise training promotes SIRT1 activity in aged rats. Rejuvenation Res. 2008; 11:139–50. 10.1089/rej.2007.0576 18069916

[9] Di Emidio G, Falone S, Vitti M, D’Alessandro AM, Vento M, Di Pietro C, Amicarelli F, Tatone C. SIRT1 signalling protects mouse oocytes against oxidative stress and is deregulated during aging. Hum Reprod. 2014; 29:2006–17. 10.1093/humrep/deu160 24963165

[10] Ding YW, Zhao GJ, Li XL, Hong GL, Li MF, Qiu QM, Wu B, Lu ZQ. SIRT1 exerts protective effects against paraquat-induced injury in mouse type II alveolar epithelial cells by deacetylating NRF2 in vitro. Int J Mol Med. 2016; 37:1049–58. 10.3892/ijmm.2016.2503 26935021

[11] Baird L, Dinkova-Kostova AT. The cytoprotective role of the Keap1-Nrf2 pathway. Arch Toxicol. 2011; 85:241–72. 10.1007/s00204-011-0674-5 21365312

[12] Zou Y, Hu M, Lee J, Nambiar SM, Garcia V, Bao Q, Chan JY, Dai G. Nrf2 is essential for timely M phase entry of replicating hepatocytes during liver regeneration. Am J Physiol Gastrointest Liver Physiol. 2015; 308:G262–68. 10.1152/ajpgi.00332.2014 25524062 PMC4329475

[13] Zou Y, Hu M, Bao Q, Chan JY, Dai G. Nrf2 participates in regulating maternal hepatic adaptations to pregnancy. J Cell Sci. 2013; 126:1618–25. 10.1242/jcs.118109 23418358 PMC3647438

[14] Deshmukh P, Unni S, Krishnappa G, Padmanabhan B. The Keap1-Nrf2 pathway: promising therapeutic target to counteract ROS-mediated damage in cancers and neurodegenerative diseases. Biophys Rev. 2017; 9:41–56. 10.1007/s12551-016-0244-4 28510041 PMC5425799

[15] Bhakkiyalakshmi E, Sireesh D, Rajaguru P, Paulmurugan R, Ramkumar KM. The emerging role of redox-sensitive Nrf2-Keap1 pathway in diabetes. Pharmacol Res. 2015; 91:104–14. 10.1016/j.phrs.2014.10.004 25447793

[16] Ma R, Li H, Zhang Y, Lin Y, Qiu X, Xie M, Yao B. The toxic effects and possible mechanisms of Brusatol on mouse oocytes. PLoS One. 2017; 12:e0177844. 10.1371/journal.pone.0177844 28542354 PMC5436816

[17] Park CE, Kim YH, Jeon EH, Cha KY, Lee SH, Lee KA. Expression of wee1 and its related cell cycle components in mouse early stage follicles. Cells Tissues Organs. 2004; 177:221–28. 10.1159/000080135 15459478

[18] Liu F, Rothblum-Oviatt C, Ryan CE, Piwnica-Worms H. Overproduction of human Myt1 kinase induces a G2 cell cycle delay by interfering with the intracellular trafficking of Cdc2-cyclin B1 complexes. Mol Cell Biol. 1999; 19:5113–23. 10.1128/MCB.19.7.5113 10373560 PMC84354

[19] Chou ST, Yen YC, Lee CM, Chen MS. Pro-apoptotic role of Cdc25A: activation of cyclin B1/Cdc2 by the Cdc25A C-terminal domain. J Biol Chem. 2010; 285:17833–45. 10.1074/jbc.M109.078386 20368335 PMC2878547

[20] Lewis KN, Wason E, Edrey YH, Kristan DM, Nevo E, Buffenstein R. Regulation of Nrf2 signaling and longevity in naturally long-lived rodents. Proc Natl Acad Sci USA. 2015; 112:3722–27. 10.1073/pnas.1417566112 25775529 PMC4378420

[21] Tomobe K, Shinozuka T, Kuroiwa M, Nomura Y. Age-related changes of Nrf2 and phosphorylated GSK-3β in a mouse model of accelerated aging (SAMP8). Arch Gerontol Geriatr. 2012; 54:e1–7. 10.1016/j.archger.2011.06.006 21784539

[22] Suh JH, Shenvi SV, Dixon BM, Liu H, Jaiswal AK, Liu RM, Hagen TM. Decline in transcriptional activity of Nrf2 causes age-related loss of glutathione synthesis, which is reversible with lipoic acid. Proc Natl Acad Sci USA. 2004; 101:3381–86. 10.1073/pnas.0400282101 14985508 PMC373470

[23] Tsakiri EN, Sykiotis GP, Papassideri IS, Gorgoulis VG, Bohmann D, Trougakos IP. Differential regulation of proteasome functionality in reproductive vs. somatic tissues of Drosophila during aging or oxidative stress. FASEB J. 2013; 27:2407–20. 10.1096/fj.12-221408 23457214 PMC4050428

[24] Chapple SJ, Siow RC, Mann GE. Crosstalk between Nrf2 and the proteasome: therapeutic potential of Nrf2 inducers in vascular disease and aging. Int J Biochem Cell Biol. 2012; 44:1315–20. 10.1016/j.biocel.2012.04.021 22575091

[25] Hu X, Roberts JR, Apopa PL, Kan YW, Ma Q. Accelerated ovarian failure induced by 4-vinyl cyclohexene diepoxide in Nrf2 null mice. Mol Cell Biol. 2006; 26:940–54. 10.1128/MCB.26.3.940-954.2006 16428448 PMC1347017

[26] Leung L, Kwong M, Hou S, Lee C, Chan JY. Deficiency of the Nrf1 and Nrf2 transcription factors results in early embryonic lethality and severe oxidative stress. J Biol Chem. 2003; 278:48021–29. 10.1074/jbc.M308439200 12968018

[27] Liu Y, Liu H, Cao H, Song B, Zhang W, Zhang W. PBK/TOPK mediates promyelocyte proliferation via Nrf2-regulated cell cycle progression and apoptosis. Oncol Rep. 2015; 34:3288–96. 10.3892/or.2015.4308 26503118

[28] Ledan E, Polanski Z, Terret ME, Maro B. Meiotic maturation of the mouse oocyte requires an equilibrium between cyclin B synthesis and degradation. Dev Biol. 2001; 232:400–13. 10.1006/dbio.2001.0188 11401401

[29] Jones KT. Turning it on and off: m-phase promoting factor during meiotic maturation and fertilization. Mol Hum Reprod. 2004; 10:1–5. 10.1093/molehr/gah009 14665700

[30] Zhang L, Hou X, Ma R, Moley K, Schedl T, Wang Q. Sirt2 functions in spindle organization and chromosome alignment in mouse oocyte meiosis. FASEB J. 2014; 28:1435–45. 10.1096/fj.13-244111 24334550 PMC3929683

[31] Davinelli S, Scapagnini G, Marzatico F, Nobile V, Ferrara N, Corbi G. Influence of equol and resveratrol supplementation on health-related quality of life in menopausal women: A randomized, placebo-controlled study. Maturitas. 2017; 96:77–83. 10.1016/j.maturitas.2016.11.016 28041599

[32] Ma R, Zhang Y, Zhang L, Han J, Rui R. Sirt1 protects pig oocyte against in vitro aging. Anim Sci J. 2015; 86:826–32. 10.1111/asj.12360 25601632

[33] Zhang T, Zhou Y, Li L, Wang HH, Ma XS, Qian WP, Shen W, Schatten H, Sun QY. SIRT1, 2, 3 protect mouse oocytes from postovulatory aging. Aging (Albany NY). 2016; 8:685–96. 10.18632/aging.100911 26974211 PMC4925822

[34] Woo SR, Byun JG, Kim YH, Park ER, Joo HY, Yun M, Shin HJ, Kim SH, Shen YN, Park JE, Park GH, Lee KH. SIRT1 suppresses cellular accumulation of β-TrCP E3 ligase via protein degradation. Biochem Biophys Res Commun. 2013; 441:831–37. 10.1016/j.bbrc.2013.10.146 24211209

[35] Huang K, Chen C, Hao J, Huang J, Wang S, Liu P, Huang H. Polydatin promotes Nrf2-ARE anti-oxidative pathway through activating Sirt1 to resist AGEs-induced upregulation of fibronetin and transforming growth factor-β1 in rat glomerular messangial cells. Mol Cell Endocrinol. 2015; 399:178–89. 10.1016/j.mce.2014.08.014 25192797

[36] Potteti HR, Rajasekaran S, Rajamohan SB, Tamatam CR, Reddy NM, Reddy SP. Sirtuin 1 Promotes Hyperoxia-Induced Lung Epithelial Cell Death Independent of NF-E2-Related Factor 2 Activation. Am J Respir Cell Mol Biol. 2016; 54:697–706. 10.1165/rcmb.2014-0056OC 26465873 PMC4942191

[37] Kwak SS, Yoon JD, Cheong SA, Jeon Y, Lee E, Hyun SH. The new system of shorter porcine oocyte in vitro maturation (18 hours) using ≥8 mm follicles derived from cumulus-oocyte complexes. Theriogenology. 2014; 81:291–301. 10.1016/j.theriogenology.2013.09.028 24220361

[38] Yoon JD, Jeon Y, Cai L, Hwang SU, Kim E, Lee E, Kim DY, Hyun SH. Effects of coculture with cumulus-derived somatic cells on in vitro maturation of porcine oocytes. Theriogenology. 2015; 83:294–305. 10.1016/j.theriogenology.2014.09.025 25442018

[39] Wang Q, Chi MM, Moley KH. Live imaging reveals the link between decreased glucose uptake in ovarian cumulus cells and impaired oocyte quality in female diabetic mice. Endocrinology. 2012; 153:1984–89. 10.1210/en.2011-1815 22294751 PMC3320263

[40] Fu W, Chen H, Wang G, Luo J, Deng Z, Xin G, Xu N, Guo X, Lei J, Jiang Q, Zhang C. Self-assembly and sorting of acentrosomal microtubules by TACC3 facilitate kinetochore capture during the mitotic spindle assembly. Proc Natl Acad Sci USA. 2013; 110:15295–300. 10.1073/pnas.1312382110 24003142 PMC3780888

