# Supplementary material for: Sirt1/Nrf2 pathway is involved in oocyte aging by regulating Cyclin B1
Source: Aging (Albany NY). 2018 Oct 27;10(10):2991–3004. doi: 10.18632/aging.101609 (PMC6224227; doi:10.18632/aging.101609)
Supplement: Supplementary Figures [file aging-10-101609-s001.pdf]

SUPPLEMENTARY FIGURES

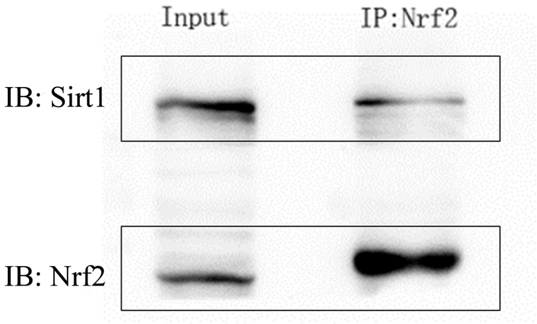

Figure S1. The CO-IP of Nrf2 and Sirt1 in mouse ovarian tissues. Ovarian tissue extracts were immunoprecipitated with anti-Nrf2 protein antibody and immunoblots were probed with anti-Sir1 antibody. The “input” panel shows target proteins prior to immuno-precipitation in the extracts.

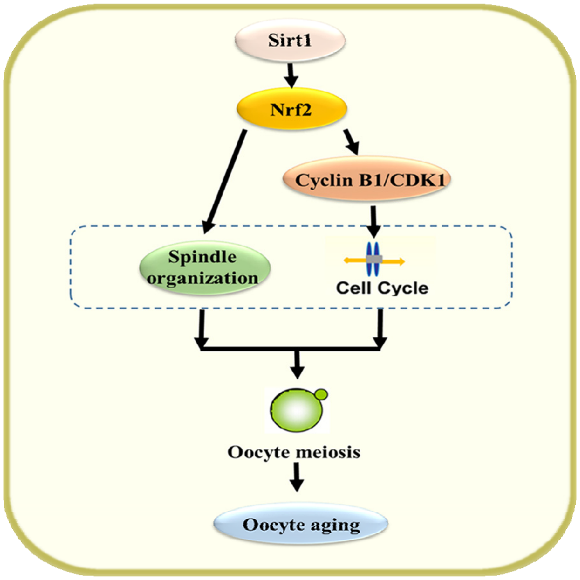

Figure S2. Summary of Nrf2-related events during mouse oocyte maturation. Nrf2 can control polar body extrusion by organizing microtubules and cell cycle that is regulated via the Sirt1-Nrf2-Cyclin B1 signaling pathway.

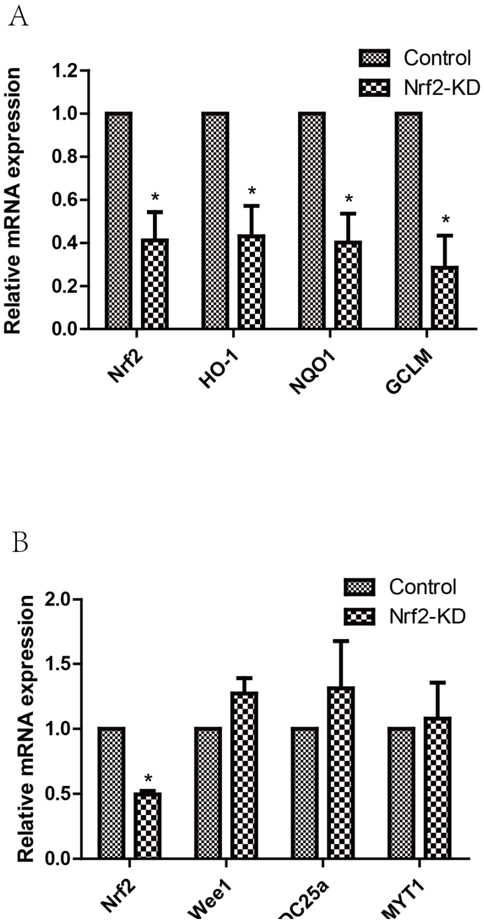

Figure S3. Effects of Nrf2 knockdown on downstream genes. (**A**) Nrf2 knockdown causes the reduction of HO-1, NQO-1 and GCLM. (**B**) No significant difference in the expression of Wee1, CDC25A and MYT1 between control and Nrf2-KD oocytes. Bars represent the mean ± SD. *P<0.05 vs. controls.
