# Supplementary material for: Sirt1/Nrf2 pathway is involved in oocyte aging by regulating Cyclin B1
Source: Aging (Albany NY). 2018 Oct 27;10(10):2991–3004. doi: 10.18632/aging.101609 (PMC6224227; doi:10.18632/aging.101609)
Supplement: Supplementary Tables [file aging-10-101609-s002.pdf]

SUPPLEMENTARY TABLES

Table S1. siRNA sequence.

	siRNA Sequence of Nrf2
**Nrf2-siRNA-1**	5' CCGAAUUACAGUGUCUUAATT 3'
	5' UUAAGACACUGUAAUUCGGTT 3'
**Nrf2-siRNA-2**	5' CGAGAAGUGUUUGACUUUATT 3'
	5' UAAAGUCAAACACUUCUCGTT 3'
**Nrf2-siRNA-3**	5' GCACAAUGGAAUUCAAUGATT 3'
	5' UCAUUGAAUUCCAUUGUGCTT 3'
	siRNA Sequence of Sirt1
**Sirt1-siRNA-1**	5' GCGGAUAGGUCCAUAUACUTT 3'
	5' AGUAUAUGGACCUAUCCGCTT 3'
**Sirt1-siRNA-2**	5' CCGUCUCUGUGUCACAAAUTT 3'
	5' AUUUGUGACACAGAGACGGTT 3'
**Sirt1-siRNA-3**	5' GGGAUCAAGAGGUUGUUAATT 3'
	5' UUAACAACCUCUUGAUCCCTT 3'

Table S2. Primer sequences.

Mouse rimer sequences		
Gene	Forward primer	Reverse primer
Nrf2	GTCTTCACTGCCCCTCATC	TCGGGAATGGAAAATAGCTCC
GAPDH	TCTTGCTCAGTGTCCTTGC	CTTTGTCAAGCTCATTTCCTGG
Sirt1	CTCTGAAAGTGAGACCAGTAGC	TGTAGATGAGGCAAAGGTTCC
Cyclin B1	CTGACCCAAACCTCTGTAGTG	CCTGTATTAGCCAGTCAATGAGG
CDK1	ACAAAGGAACAATCAAACTGGC	AGCAACACTTCTGGAGATCG
Keap1	CTCCGCAGAATGTTACTATCCAG	ACACTGTTCAACTGGTCCTG
Nrf1	AATGTCCGCAGTGATGTCC	GCCTGAGTTTGTGTTTGCTG
Human primer sequences		
Gene	Forward primer	Reverse primer
Nrf2	AAACCACCCTGAAACGACAG	AGCGGCTTGAATGTTTGTC
GAPDH	ACATCGCTCAGACACCATG	TGTAGTTGAGGTCAATGAAGGG
